# Pediatric gastroenteritis in the emergency department: practice evaluation in Belgium, France, The Netherlands and Switzerland

**DOI:** 10.1186/1471-2431-14-125

**Published:** 2014-05-16

**Authors:** Raphaëlle Pelc, Sébastien Redant, Sébastien Julliand, Juan Llor, Mathie Lorrot, Rianne Oostenbrink, Vincent Gajdos, François Angoulvant

**Affiliations:** 1 Department General Pediatrics, CHI de Creteil, Creteil, France; 2 Pediatric Emergency Department, Queen Fabiola Hospital, Brussels, Belgium; 3 Department of Pediatric Emergency, AP-HP, Hôpital Robert Debré, Université Paris Diderot, Sorbonne Paris Cité, Paris, France; 4 Department of Medical and Surgical Pediatric, Hôpital du Valais, Centre Hospitalier du Valais Romand, Sion, Switzerland; 5 Department General Pediatrics, AP-HP, Hôpital Robert Debré, Université Paris Diderot, Sorbonne Paris Cité, Paris, France; 6 Department General Pediatrics, Erasmus MC-Sophia Hospital, Rotterdam, Netherlands; 7 Pediatric Department, AP-HP, Hôpital Antoine Béclère and Université Paris 11, Clamart, France; 8 Inserm, CESP Centre for Research in Epidemiology and Population Health, U1018, Reproduction and Child Development Team, Villejuif, France; 9 Department of Pediatric Emergency, AP-HP, Hôpital Necker-Enfants Malades, Université Paris Descartes, Sorbonne Paris Cité, 149 rue de Sèvres, 75015 Paris, France

**Keywords:** Acute gastroenteritis, Pediatric emergency department, Practice patterns, Rehydration

## Abstract

**Background:**

Based on European recommendations of ESPGHAN/ESPID from 2008, first line therapy for dehydration caused by acute gastroenteritis (AGE) is oral rehydration solution (ORS). In case of oral route failure, nasogastric tube enteral rehydration is as efficient as intra-venous rehydration and seems to lead to fewer adverse events. The primary objective was to describe rehydration strategies used in cases of AGE in pediatric emergency departments (PEDs) in Belgium, France, The Netherlands, and Switzerland.

**Methods:**

An electronic survey describing a scenario in which a toddler had moderate dehydration caused by AGE was sent to physicians working in pediatric emergency departments. Analytical data were analyzed with descriptive statistics and Kruskal –Wallis Rank test.

**Results:**

We analyzed 68 responses, distributed as follows: Belgium N = 10, France N = 37, The Netherlands N = 7, and Switzerland N = 14. Oral rehydration with ORS was the first line of treatment for 90% of the respondents. In case of first line treatment failure, intravenous rehydration was preferred by 95% of respondents from France, whereas nasogastric route was more likely to be used by those from Belgium (80%), The Netherlands (100%) and Switzerland (86%). Serum electrolyte measurements were more frequently prescribed in France (92%) and Belgium (80%) than in The Netherlands (43%) and Switzerland (29%). Racecadotril was more frequently used in France, and ondansetron was more frequently used in Switzerland. No respondent suggested routine use of antibiotics.

**Conclusion:**

We found variations in practices in terms of invasiveness and testing. Our study supports the need for further evaluation and implementation strategies of ESPGHAN/ESPID guidelines. We plan to extend the study throughout Europe with support of the Young ESPID Group.

## Background

Acute gastroenteritis (AGE) in children is very common and accounts for a large number of emergency department visits and hospitalizations [1]. The most dangerous complication is dehydration, and every year, there are at least 230 deaths and over 87,000 hospitalizations of children under 5 years of age in the European Union [2]. In Europe, incidence of AGE range from 0.5 to 1.9 episodes per year per person, with a higher risk for children under 3 years [3]. The management of children diagnosed with AGE is based largely upon international recommendations. The latest European recommendations from European Society for Paediatric Gastroenterology Hepatology and Nutrition/European Society for Paediatric Infectious Disease (ESPGHAN/ESPID) published in 2008 [3,4], specify preferred methods of rehydration, possible medications, potentially useful laboratory tests, and suggested nutrition in cases of AGE. These recommendations clearly state that the first line of treatment should include oral rehydration with standard Oral Rehydration Solution (ORS), the composition of which is specified within the same recommendations [3]. In contrast, treatment recommendations are less strict regarding second line treatments. Indeed, recommendations indicate that the nasogastric (NG) and intravenous (IV) routes can both be used to rehydrate individuals with AGE even though the recommendations additionally state that the NG route is associated with less adverse events and shorter hospitalizations [5,6]. They also clearly state that there is no need for microbiological investigation since the epidemiology of AGE is well known in Europe [3]. These recommendations, however, do not take into account the most recent studies on AGE treatments, such as the study of ondansetron by Carter *et al.*[7]. Where guidelines are vague or evidence is limited, wide variations in the management of AGE have been observed among institutions and countries [8,9]. Because an accurate understanding of current treatment regimens is a necessary prerequisite to developing improvements, we sought to assess variations in the management of pediatric AGE across Europe.

Our primary objective was to determine the extent to which significant variations in rehydration therapies for individuals with pediatric gastroenteritis exist among pediatric emergency departments (PEDs) in Europe. Secondary objectives included the assessment of variations in the use of additional therapeutic and diagnostic modalities.

## Methods

### Study design

This study is a cross-sectional electronic survey of physicians regarding their management of pediatric AGE. Participants included practicing physicians within PEDs of teaching hospitals in Belgium, France, The Netherlands and Switzerland.

### Population

We chose to conduct the study in those 4 countries due to their geographical and linguistic proximity. We selected primarily teaching hospitals because smaller hospitals often consider those facilities as reference sources. Our survey was sent to both senior, junior physicians and residents. Every center was asked to include at least 3 participants to improve the measurement representativeness. All participants who responded were included in the analyses. An initial power analysis determined that at least 40 centers should be included with 3 physicians per center (120 participants).

### Survey

Following the recommendations from Burns *et al.*, we performed a literature review and consulted an expert panel to assist in item generation to create a self-report questionnaire containing 24 items [10]. Another panel was recruited to pretest the survey; their responses were not included in the data analysis.

The survey began with a brief scenario describing a toddler presenting with AGE and moderate dehydration (Additional file 1). Eight survey items collected demographic information, including country and city of practice, the number of year of experience in PED and the number of visits per year. Additional survey items included questions about treatments for AGE in PEDs. Different types of response modalities were utilized, including dichotomous questions (yes/no) and questions addressing the frequency of endorsement with numeric response options (<5%, 5-30%, 31-69%, 70-95%, >95%). The items were independent and non-compulsory.

### Procedure

The study was conducted between February and July 2012. The survey was emailed to participants following a phone contact to increase the potential for a large number of responses and was accessible in both English and French on a dedicated website (https://sites.google.com/site/hydragast/). A reminder was sent to a non-respondent’s facility 3 weeks after sending the first e-mail. If we had only one response from a particular facility, an additional reminder was sent in hopes of acquiring other responses from the same center.

### Data analysis

Data were analyzed using the statistical software Stata (StataCorp LP, College Station, Texas, USA). To determine the preferred treatment (frequency ≥ 70%), responses were grouped by frequency of endorsement questions in two categories. This decision was driven by the distribution of the data and ease of interpretation [9]. Categorical data were analyzed using the Kruskal-Wallis rank test, and other data were analyzed via descriptive analysis, with each country being analyzed separately. Subsequently, because of potential response homogeneity, countries were clustered for analysis using the Wilcoxon rank sum test. Approval from the Ethics committee was not needed because this study is reflective of opinions more than actual practice and no real patients were included.

## Results

### Description of participants

We sent the survey to 17 centers in France, 6 in Belgium, 12 in Switzerland and 7 in The Netherlands. 68 surveys were completed and returned, and all were analyzed. The response rate when we compare the number of answers received to the number of answers expected from the power analysis is 54%. We received 37 surveys from 14 centers in France, 10 surveys from 6 centers in Belgium, 14 surveys from 6 centers in Switzerland and 7 surveys from 3 centers in The Netherlands, yielding an average of 2.3 responses per center (range = 1 to 6). There were 7 juniors and 61 seniors. Table 1 includes item results relative to the frequency of endorsement.

**Table 1:** Preferential practices regarding management of pediatric acute gastroenteritis

**Preferential practices (>70%)**	**Total**	**Belgium**	**The Netherlands**	**Switzerland**	**France**
	**N = 68**	**N = 10**	**N = 7**	**N = 14**	**N = 37**
	**CI 95%**				
First-intention rehydration method					
ORS oral route	61 (90%)	10 (100%)	5 (71%)	12 (86%)	34 (92%)
	CI [80–96]				
ORS pure	62 (91%)	10 (100%)	6 (86%)	13 (93%)	33 (89%)
	CI [82–97]				
Rehydration route in case of oral rehydration failure					
Intra-venous route	35 (51%)	0 (0%)	0 (0%)	0 (0%)	35 (95%)
	CI [39–64]				
Nasogastric route (with ORS)	28 (41%)	8 (80%)	7 (100%)	12 (86%)	1 (3%)
	CI [29–54]				
Medication in case of oral rehydration failure					
Ondansetron	6 (9%)	0 (0%)	1 (14%)	5 (36%)	0 (0%)
	CI [3–18]				
Racecadotril	19 (28%)	0 (0%)	0 (0%)	0 (0%)	19 (51%)
	CI [18–40]				
Laboratory tests in case of oral rehydration failure					
Electrolytes	49 (72%)	8 (80%)	3 (43%)	4 (29%)	34 (92%)
	CI [60–82]				
Blood count	28 (41%)	6 (60%)	2 (29%)	1 (7%)	19 (51%)
	CI [29–54]				
C-reactive protein	25 (37%)	6 (60%)	2 (29%)	1 (7%)	16 (43%)
	CI [25–49]				
Stool virology	20 (29%)	5 (50%)	2 (29%)	1 (7%)	12 (32%)
	CI [19–42]				
Stool culture	11 (16%)	2 (20%)	3 (43%)	0 (0%)	6 (16%)
	CI [8–27]				

### First line rehydration therapy

Ninety percent of respondents (N = 61) reported the use of oral route ORS as their first line of rehydration therapy in children with moderate dehydration caused by infectious AGE. There was no significant difference among countries. Non-modified ORS was chosen as the primary liquid for oral rehydration by 91% (N = 62) of respondents.

### Second line rehydration therapy

In the case of oral rehydration failure, while IV rehydration was the preferred second line treatment for 95% (N = 35) of respondents in France, no respondent from the 3 other countries reported a preference for IV rehydration in such cases (*P* < 0.001; Wilcoxon rank sum test). In contrast, NG rehydration was the preferred second line treatment of respondents in Belgium, 80% (N = 8); The Netherlands, 100% (N = 7); and Switzerland, 86% (N = 12); whereas only one respondent (3%) in France reported it as his preferential treatment (*P* < 0.001; Wilcoxon rank sum test).

Results describing the composition of fluids in the case of IV rehydration were widely heterogeneous, with over 15 different combinations reported by respondents. In 4 cases, respondents reported choosing not to use standard fluid in favor of utilizing hand-made fluid adapted specifically to each patient. However, normal saline (0.9% NaCl) was the most frequently used fluid reported (N = 10/27, 37%) in Belgium, The Netherlands and Switzerland. In France, 56% of the participants (N = 18/32) reported frequently using a fluid composed of 5% glucose with 4 g/L NaCl and 2 g/L KCl. The volume of fluid administered during the first 4 hours in cases of IV rehydration was also widely heterogeneous, with responses ranging from 10 mL/kg to 100 mL/kg and a median of 15 mL/kg.

### Laboratory testing

80% (N = 8) of respondents from Belgium, 92% (N = 34) of respondents from France, 43% (N = 3) of respondents from The Netherlands and 29% (N = 4) of respondents from Switzerland, conducted tests for serum electrolyte in more than 70% of the time. In contrast, patient’s stool was tested for viruses more than 70% of the time by only 29% (N = 20) of the respondents, and stool cultures were performed by 16% (N = 11) of the respondents. A blood count and/or C-reactive protein was performed by 46% (N = 31) of respondents; only 4 respondents reported testing blood count only, and one respondent reported testing C-reactive protein only. Other laboratory tests reported by the participants, but not listed in our questionnaire, included a urine stick test, tests for ketonemia, abdominal ultrasonography and an arterial blood gas test.

### Drug prescription

Antiemetic agents, such as ondansetron, metoclopramide, domperidone, were rarely reported to be prescribed according to respondents. Among those drug types, ondansetron was reported the most frequently, by 9% (N = 6) of respondents, most of whom were from Switzerland (N = 5). No respondent reported the use of antimotility (loperamide) drugs. Probiotics were reported as prescribed more than 70% of the time by only one respondent. Fifty-one percent (N = 19) of the respondents from France reported prescribing an antisecretory drug (racecadotril) more than 70% of the time, but no such use was reported by physicians in the other countries (P < 0.001; Wilcoxon rank sum test). Antibiotics were reported as never prescribed by 87% (N = 59) of respondents. None of the respondent reported the preferential use of adsorbent (smectite).

### Nutrition

Survey reports of food withdrawal duration varied from 2 hours to 24 hours, with a median of 6 hours.

## Discussion

Our study is the first to use a self-report questionnaire to assess and compare physician practice patterns in the treatment of pediatric AGE in European PEDs. The results suggest that the first line of rehydration therapy recommendations are well known, with the use of oral rehydration with ORS reported by 90% of the respondents, without variation, across Belgium, France, The Netherlands and Switzerland. These frequencies are larger than those reported by Freedman *et al.* in North-America: only 76% of Canadian physicians and 46% of Americans reported oral rehydration as their first line of rehydration therapy [9].

Wide practice variations were observed for second line rehydration treatments and for the type and volume of fluid reported for IV rehydration. This finding reflects the variability of European recommendations on this subject because two equivalent rehydration routes were reported [3]. However, less within-country variability in the type of IV or NG rehydration was observed, suggesting an influence of training and health care organization, specific to each country, on physician practices [9]. Hoekstra in Australia and New-Zealand [11], and Karpas in Canada [12], have also shown differences in practice after ORS failure in different hospitals within the same country.

Among the four countries examined for this study, respondents from France were the ones who most often chose the IV route and ordered serum electrolyte testing, a finding possibly explained by the recommendation to monitor IV rehydration [3]. Microbiological examinations were commonly reported in our study even though these exams are not routinely recommended for children with AGE [3]. Few drugs were reported to be frequently prescribed, and these varied across countries. Despite the lack of recommendations, the use of racecadotril was frequently reported by French respondents, whereas the use of ondansetron was reported often by Swiss respondents. The recommendations concerning laboratory testing and medication are maybe less known than the ones concerning the rehydration.

Overall, our results suggest that interventions to increase the homogeneity of practices in the management of pediatric AGE could be useful [13], especially regarding adjuvant therapy such as racecadotril use and laboratory testing. Similarly, in light of the benefits of NG rehydration in terms of costs and side effects, the implementation of this method should be considered in France. Despite current recommendations [3], ondansetron use was frequently reported by respondents in Switzerland. This treatment does seem to facilitate oral rehydration [14], and some evidence was not available when the European recommendations were published in 2008. Nonetheless, a real risk/benefit assessment of the widespread use of ondansetron in AGE in Europe is still lacking. Studies have shown that parents prefer IV rehydration [12] and treatments that shorten diarrhea duration [15]. With respect to health care providers, another recent study indicated that only 14% of physicians favor NG over IV rehydration [16]. These elements highlight the need to refine the current recommendations for the management of pediatric AGE to avoid unfounded practice variations.Two major issues should be redefined: to favor one treatment over the other for the second line rehydration therapy; and to update the pharmacological therapy statement, especially concerning the use of ondansetron, based on the recent evidences [7].

### Limitations

The low response rate (54%) could have introduced a self-selection bias. Likewise, the low number of respondents (68) limits the external validity of the study. Most participants worked in a teaching hospital, which may not be representative of the entire health care structures that treats children’s AGE. Additionally, this study is reflective of opinions more than actual practice patterns because it is difficult to determine what respondents actually do versus what they claim they do.

## Conclusion

We observed good adherence to the European guidelines for treating AGE in the 4 countries, especially concerning first line therapy and nutrition. However, our study highlights wide variations in second line rehydration strategies and drug prescriptions among countries. We plan to extend this study to other European countries with the help of the Young ESPID group.

## Abbreviations

AGE: Acute gastroenteritis; ESPGHAN: European society for paediatric gastroenterology hepatology and nutrition; ESPID: European society for paediatric infectious disease; IV: Intravenous; NG: Nasogastric; ORS: Oral rehydration solution; PED: Pediatric emergency departments.

## Competing interest

The authors declare that they have no competing interests.

## Authors’ contribution

FA, SR, ML, SJ, JL, RP conceived the study. FA led the protocol design process. VG, ML, RO revised the methodology. FA, RP, SR, SJ, JL, RO participated to the network and collected data. FA, VG, RO, RP performed the statistical analysis. RP make the first draft of the manuscript. All authors read, revised, and approved the final manuscript.

## Pre-publication history

The pre-publication history for this paper can be accessed here:

http://www.biomedcentral.com/1471-2431/14/125/prepub

## Supplementary Material

Additional file 1 Survey on the practices of physician in the Emergency Department to rehydrate children with acute gastroenteritis. Click here for file
