# Supplementary material for: Pediatric gastroenteritis in the emergency department: practice evaluation in Belgium, France, The Netherlands and Switzerland
Source: BMC Pediatr. 2014 May 16;14:125. doi: 10.1186/1471-2431-14-125 (PMC4045874; doi:10.1186/1471-2431-14-125)
Supplement: Additional file 1 — Survey on the practices of physician in the Emergency Department to rehydrate children with acute gastroenteritis. [file 1471-2431-14-125-S1.pdf]

Hospital

What is your role in the unit?

head of department ▼

How many years you been working in a pediatric emergency department?

How many patients under 18 years of age come annually to your emergency department?

City

Country

Belgium ▼

In the following scenario, how often do you initiate rehydration ORALLY as first-line therapy?

«You evaluate a 15-month-old child with a history and physical examination consistent with moderate dehydration. The child has had 7 episodes of nonbloody diarrhea today and 5 episodes of vomiting, with the last occurring 45 minutes ago. The presumed etiology is infectious gastroenteritis»

- <5% or never
- 5 to 30%
- 31 to 69%
- 70 to 95%
- >95% or always

In case of oral rehydration, which fluid do you use?

	< 5% or never	5 to 30%	31 to 69%	70 to 95%	> 95% or always
Oral rehydration solution (ORS)	○	○	○	○	○
Modified Oral rehydration solution (sugar,juice,water,...)	○	○	○	○	○
fruit juice	○	○	○	○	○
soda	○	○	○	○	○

Other, please precise	○	○	○	○	○

If you sometimes use other fluids, please precise the type of fluid

In the following scenario, in case of oral rehydration therapy (ORT) FAILURE which method do you use?

«You evaluate a 15-month-old child with a history and physical examination consistent with moderate dehydration. The child has had 7 episodes of nonbloody diarrhea today and 5 episodes of vomiting, with the last occurring 45 minutes ago. The presumed etiology is infectious gastroenteritis»

	< 5% or never	5 to 30%	31 to 69%	70 to 95%	> 95% or always
Intravenous rehydration	○	○	○	○	○
Enteral rehydration via the nasogastric route	○	○	○	○	○

In case of INTRAVENOUS rehydration in this situation, which fluid do you use more often during the first four hours? (commercial name)

In the 5 following questions please describe the fluid you use for intravenous rehydration

Glucose concentration of the IV solution

Glucose : G/L

Sodium concentration of the IV solution

Na+ : G/L

Potassium concentration of the IV solution

K+: G/L

Bicarbonate concentration of the IV solution

Bicar: G/L

fluid volume used in the first 4 hours

mL/kg/h

If you use ENTERAL rehydration via the nasogastric route, which solution do you use ? if you don't, do not respond

- [ ] Oral rehydration solution
- [ ] Autre :

If you use an other solution, please precise

In the following scenario, in case of Oral rehydration therapy failure, how often do you realize those tests?

«You evaluate a 15-month-old child with a history and physical examination consistent with moderate dehydration. The child has had 7 episodes of nonbloody diarrhea today and 5 episodes of vomiting, with the last occurring 45 minutes ago. The presumed etiology is infectious gastroenteritis»

	< 5% or never	5 to 30%	31 to 69%	70 to 95%	> 95% or always
blood count	○	○	○	○	○
serum electrolytes (Na, K, Cl, Bicarbonate, Urea)	○	○	○	○	○
Glycemia	○	○	○	○	○
CRP	○	○	○	○	○
Procalcitonin	○	○	○	○	○
blood culture	○	○	○	○	○
stool culture	○	○	○	○	○
stool virology	○	○	○	○	○
stool parasitology	○	○	○	○	○
other	○	○	○	○	○

In the following scenario, in case of Oral rehydration therapy failure, how often do you initiate a pharmacological treatment ? and which one do you use ?

« You evaluate a 15-month-old child with a history and physical examination consistent with moderate dehydration. The child has had seven episodes of nonbloody diarrhea today and five episodes of vomiting, with the last occurring 45 minutes ago. The presumed etiology is infectious gastroenteritis »

	< 5% or never	5 to 30%	31 to 69%	70 to 95%	> 95% or always
Ondansetron	○	○	○	○	○
Metoclopramide	○	○	○	○	○

Domperidone	○	○	○	○	○
Loperamide	○	○	○	○	○
Smectite	○	○	○	○	○
Racecadotril	○	○	○	○	○
Antibiotic	○	○	○	○	○
Lactobacillus GG	○	○	○	○	○
Saccharomyces boulardii	○	○	○	○	○
other treatment or other probiotic	○	○	○	○	○

If you use an other treatment, please precise which one

In case oral rehydration therapy failure, how long do you wait (in hours) before restarting feeding ? (ORS excluded)

Envoyer

N'envoyez jamais de mots de passe via Google Formulaires.

Fourni par Google Documents

Signaler un cas d'utilisation abusive - Conditions d'utilisation - Clauses additionnelles
